# Oxidized LDL Modifies the Association between Proteinuria and Deterioration of Kidney Function in Proteinuric Diabetic Kidney Disease

**DOI:** 10.3390/life11060504

**Published:** 2021-05-29

**Authors:** Stefanos Roumeliotis, Panagiotis I. Georgianos, Athanasios Roumeliotis, Theodoros Eleftheriadis, Aikaterini Stamou, Vangelis G. Manolopoulos, Stylianos Panagoutsos, Vassilios Liakopoulos

**Affiliations:** 1 Division of Nephrology and Hypertension, 1st Department of Internal Medicine, AHEPA Hospital, School of Medicine, Aristotle University of Thessaloniki, 54636 Thessaloniki, Greece; pangeorgi@yahoo.gr (P.I.G.); a_roumeliotis@hotmail.com (A.R.); liakopul@otenet.gr (V.L.); 2 Department of Nephrology, School of Medicine, University of Thessaly, 38221 Larissa, Greece; teleftheriadis@yahoo.com; 3 Department of Microbiology, AHEPA Hospital, School of Medicine, Aristotle University of Thessaloniki, 54636 Thessaloniki, Greece; katerina_stms@yahoo.gr; 4 Laboratory of Pharmacology, Medical School, Democritus University of Thrace, 68100 Alexandroupolis, Greece; emanolop@med.duth.gr; 5 Department of Nephrology, Medical School, Democritus University of Thrace, 68100 Alexandroupolis, Greece; spanagou@med.duth.gr

**Keywords:** diabetic kidney disease, effect modification, eGFR, oxidized LDL, oxLDL, oxidative stress, proteinuria

## Abstract

Proteinuria is characterized by low accuracy for predicting onset and development of diabetic kidney disease (DKD) because it is not directly associated with molecular changes that promote DKD, but is a result of kidney damage. Oxidized low-density lipoprotein (ox-LDL) reflects oxidative stress and endothelial dysfunction, both underlying the development of proteinuria and loss of kidney function in DKD. We aimed to investigate whether ox-LDL modifies the association between proteinuria and progression of DKD in a cohort of 91 patients with proteinuric DKD and diabetic retinopathy, followed for 10 years. The primary endpoint was a combined kidney outcome of eGFR decline ≥30% or progression to end-stage kidney disease. After the end of the study, we considered the percentage change of eGFR over time as our secondary outcome. Proteinuria was associated with both outcomes, and ox-LDL amplified the magnitude of this link (*p* < 0.0001 for primary and *p* < 0.0001 for secondary outcome, respectively). After adjustment for duration of diabetes, history of cardiovascular disease and serum albumin, ox-LDL remained a significant effect modifier of the association between proteinuria and eGFR decline over time (*p* = 0.04). Our study shows that in proteinuric DKD, circulating ox-LDL levels amplified the magnitude of the association between proteinuria and progression of DKD.

## 1. Introduction

During the past decade, the prevalence of type 2 diabetes mellitus (T2DM) has increased to epidemic proportions worldwide. One of the most common complications of T2DM, diabetic kidney disease (DKD), occurs in 40% of T2DM patients over their lifetime and is the main cause of end-stage kidney disease (ESKD) in the USA and worldwide [1]. In recent years it became evident that, besides its traditional proteinuric form, DKD could also progress through a non-proteinuric pattern, depicting DKD as a heterogenous disease with highly complex pathophysiology [2]. Compared to non-proteinuric, proteinuric DKD patients manifest faster progression to ESKD, indicating that proteinuria could be an independent factor associated with deterioration of kidney function. However, proteinuria is characterized by significant variability and decreased accuracy for predicting onset and development DKD, probably because it is not directly associated with molecular changes that promote DKD, per se, but is a result of kidney damage. On the other hand, oxidative stress (OS) and endothelial dysfunction (ED) have been widely recognized as pathogenetic mechanisms underlying DKD. The hyperglycemia-derived imbalance between antioxidants and pro-oxidants in favor of the latter, termed as OS, occurs even at early stages of DKD, is gradually increased as DKD progresses, is directly associated with declining estimated glomerular filtration rate (eGFR), and is even more pronounced in ESKD [3,4,5]. OS along with inflammation trigger the oxidative modification of low-density lipoprotein (LDL) within the vessel wall, to form oxidized LDL (ox-LDL), a highly reactive molecule which serves as a marker of both OS and ED status [6]. In experimental uremia, OS has been tightly linked with inflammation and atherosclerosis [7], whereas, when human macrophages are exposed to high ox-LDL levels, they release anti-inflammatory molecules, as a defense mechanism. Increased uptake of ox-LDL by macrophages upregulates the formation and function of various biomarkers of inflammation and OS [8]. The interplay between ox-LDL and inflammatory cytokines has been also verified by clinical data [9]. Both experimental and clinical data report that ox-LDL promotes glomerular and tubulointerstitial injury and damage in podocytes, resulting in development of proteinuria and loss of kidney function. Therefore, it is suggested that ox-LDL might play an important role in the pathogenesis of proteinuric DKD. ox-LDL has also been repeatedly associated with the presence and development of proteinuria in DKD [10,11,12]. However, to date, the interplay between ox-LDL and proteinuria has been poorly described in DKD patients. Since ox-LDL is a marker of OS and ED that promotes development of DKD and proteinuria, in this study, we aimed to investigate whether ox-LDL might modify the association between proteinuria and deterioration of kidney function in a cohort of patients with established proteinuric DKD.

## 2. Materials and Methods

### 2.1. Patients

We enrolled 91 patients with documented proteinuric DKD, followed in the Diabetic Nephropathy Clinic of the University General Hospital of Alexandroupolis, Greece. To establish proteinuric DKD, and not T2DM and chronic kidney disease (CKD) from another cause, inclusion criteria included history of documented T2DM for at least 7 years, presence of diabetic retinopathy, eGFR < 90 mL/min and persistent proteinuria, whereas patients with acute illness, tumor, chronic inflammation, urinary tract disease or any evidence of non-diabetic CKD were excluded from the study. At baseline we recorded demographic, somatometric and clinical data, and we obtained blood samples from all participants. eGFR was calculated by the CKD-EPI calculation.

### 2.2. Study Protocol

Both the PhD Scientific Committee and the Ethics Committee of the Scientific Council of the University General Hospital of Alexandroupolis approved our study protocol in 2007 (1046/1 November 2007) and re-approved it after a minor correction regarding the calculation of eGFR in 2011 (1130/25 November 2011). Our study protocol was in conformity with the Helsinki Declaration of Human Rights. All participants provided written informed consent at enrollment.

### 2.3. Laboratory Analyses

Laboratory analyses have been extensively described before [13]. In short, at baseline and after the study period we collected whole blood, serum, plasma and spot urine samples from all patients. Proteinuria and eGFR were evaluated both at baseline and after the study period. Proteinuria was assessed as the ratio of urine protein to creatinine (UPCR) in 3 consecutive morning spot samples, and plasma ox-LDL levels were determined by the ELISA sandwich antibodies method (human oxidized LDL ELISA kit, Mercodia, Sweden) as described elsewhere [14].

### 2.4. Follow-up

After initial assessment, all patients were prospectively followed up for 10 years (2008–2018) with the primary outcome being the progression to ESKD requiring dialysis or eGFR decline of at least 30% from baseline. Death was accurately recorded and was considered as a competitive event. To determine the deterioration of kidney function after the follow-up, we re-evaluated eGFR at the end of the study, and we calculated the percentage eGFR change over time (ΔeGFR/baseline eGFR divided by the follow-up time) for each patient (secondary endpoint). We collected follow-up data for all participants via follow-up visits, hospital medical records, death certificates or via integrated telephone interview.

### 2.5. Statistics

We used the Shapiro–Wilk test to test data for normality, and normally distributed variables were presented as mean (SD), whereas non-normally variables were presented as median (range) and binary variables as percent frequency. To identify potential confounders associated with deterioration of kidney function, and possibly acting on the effect modification by ox-LDL on the UPCR-deterioration of kidney function association, we divided our study cohort into tertiles according to eGFR values (18–41.3, 42–72.4, 73.4–89.6 mL/min), Table 1. Differences of all baseline variables among eGFR tertiles were explored using the Mann–Whitney test for continuous and chi-square test for categorical variables. We modeled the effect modification by ox-LDL cholesterol on the association between UPCR and the primary kidney outcome, by performing univariate and multivariate Fine and Gray regression models, which have been used before and take into account the competitive risk of death [15,16,17]. In the univariate analysis we tested all variables in Table 1 and identified ox-LDL, UPCR, their interaction term (ox-LDLxUPCR), baseline eGFR, systolic blood pressure (SBP), serum albumin and duration of T2DM as predictors of the primary kidney outcome of eGFR decline ≥30% or progression to ESKD requiring dialysis. On the multivariate analysis we included all these variables that were associated with the primary outcome.

For the secondary endpoint, we forced all baseline variables from Table 1 with the percentage change of eGFR over time as the dependent variable in a stepwise multiple regression analysis. In univariate regression models, ox-LDL, UPCR, ox-LDLxUPCR, serum albumin, duration of T2DM and history of cardiovascular (CV) disease were associated with the change of eGFR. Multivariate regression models included all these markers as independent variables. We used the standard linear combination method to perform the effect modification analysis. Data were presented as sub-hazard ratios (SHRs), *p* values and 95% confidence intervals (CIs). Statistical analyses were conducted by STATA 13.0 for Windows, College Station, Texas, USA and IBM Statistical package for Social Sciences (SPSS 18.0 for Window, Chicago, IL, USA).

## 3. Results

### 3.1. Baseline Characteristics

Baseline anthropometric, clinical and biochemical data of 91 patients with proteinuric DKD in total and according to eGFR tertiles are presented in Table 1. The study population included 91 patients (median age 67 years (47–84), males 47%) with T2DM for a median of 13 years and median eGFR 59.6 mL/min. Approximately 7 out of 10 had background history of CV disease, and patients were mainly overweight and obese with a mean waist circumference of 106.4 cm, but had acceptable control of glycemia and hypertension, assessed by a median SBP/diastolic blood pressure (DBP) of 140/80 mm Hg and a median glycated hemoglobin (HbA1c) of 7.2%. The mean values of CRP ± SD in eGFR tertiles 1, 2 and 3 were 0.56 ± 0.6, 0.59 ± 1.9 and 0.36 ± 0.9 mg/dL, respectively. There was no significant difference in the proportion of sexes, history of CV events and glycemic control across tertiles. Compared to the third, patients in the first tertile (with the lowest values of eGFR) had significantly increased age, duration of T2DM, SBP, triglycerides and C-reactive protein (CRP) levels. As eGFR decreased, there was a significant, graded decrease in serum albumin and hemoglobin levels, whereas UPCR and ox-LDL progressively increased from the third to the first tertile. ANOVA test showed that between tertiles 1 and 2, UPCR, hemoglobin, duration of T2DM and serum albumin were significantly different; tertiles 2–3 differed significantly in UPCR, age, ox-LDL, SBP and triglycerides, whereas UPCR, SBP, ox-LDL, age, hemoglobin, duration of hypertension and T2DM and HDL cholesterol were significantly different among tertiles 1–3.

### 3.2. Outcomes

**Figure 1:**
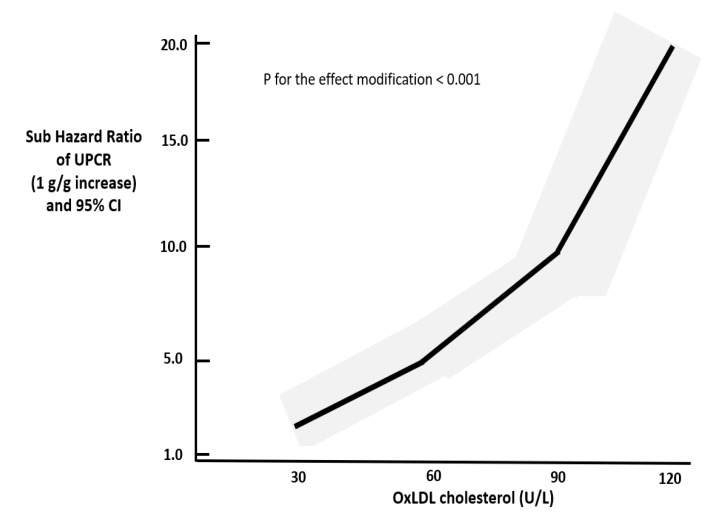
Ox-LDL as effect modifier of the association between UPCR and progression of DKD. The black continuous line represents the shape of sub-hazard ratios throughout various levels of ox-LDL, and the gray areas correspond to 95% CI (*p* for effect modification < 0.001).

During the follow-up period (median 89 months; range 23–120 months, total person-time: 7569 months), 17 patients presented the combined kidney outcome of eGFR decline of at least 30% from baseline or progression to ESKD, whereas 13 patients died. On univariate Fine and Gray analysis, baseline UPCR and plasma ox-LDL were significantly associated with the composite kidney endpoint (SHR 1.53, 95%CI 1.15–2.03, *p* = 0.003 and 1.05, 95%CI 1.02–1.08, *p* < 0.001, respectively). To find possible determinants of deterioration of kidney function, we performed Fine and Gray regression analysis on an exploratory basis for all variables in Table 1, and we found that only baseline eGFR (SHR 0.97, 95%CI 0.95–0.99, *p* = 0.032), SBP (SHR 0.96, 95%CI 0.93–0.99, *p* = 0.01), T2DM duration (SHR 1.06, 95%CI 1.00–1.11, *p* = 0.03) and serum albumin (SHR 0.25, 95%CI 0.11–0.60, *p* = 0.002) were associated with the composite outcome. Fine and Gray analysis showed that ox-LDL cholesterol was a strong modifier of the association between UPCR and the composite outcome of kidney function deterioration in the crude analysis (SHR 1.01, 95%CI 1.00–1.01, *p* < 0.001), Table 2 and Figure 1. In the multivariate analysis, adjusted for the variables that were associated with the study outcome in univariate models, only ox-LDL (SHR 1.07, 95%CI 1.03–1.12, *p* = 0.002) and SBP (SHR 0.97, 95%CI 0.94–0.99, *p* = 0.005) remained significant, independent predictors of eGFR decline of at least 30% from baseline or progression to ESKD.

Figure 1 shows that the unfavorable effect for eGFR decline portended by a fixed increase in baseline UPCR (1 g/g) was progressively higher across increasing values of ox-LDL cholesterol. As ox-LDL increased, every 1 g/g UPCR was associated with more increased danger for developing the composite kidney endpoint.

**Figure 2:**
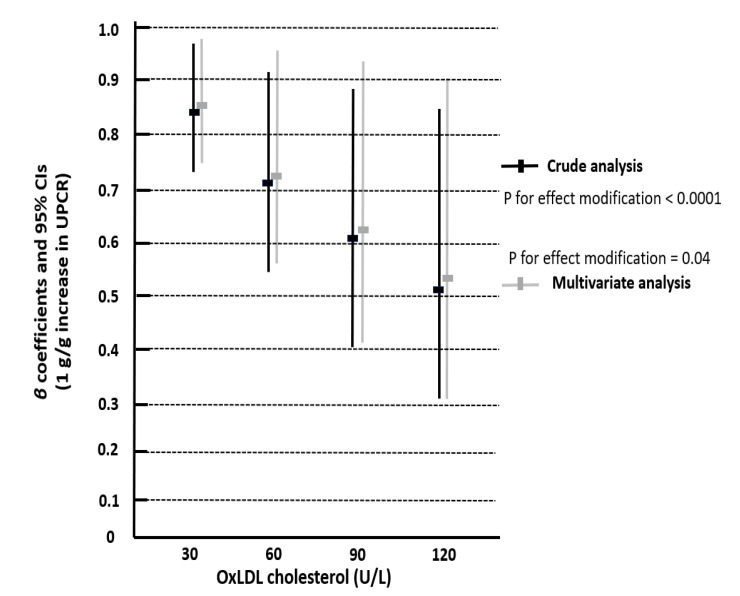
Effect modification by ox-LDL on UPCR decline in eGFR over time, in crude (*p* for effect modification < 0.001) and multivariate analyses adjusted for serum albumin, triglycerides, duration of T2DM and history of CV disease (*p* for effect modification = 0.04).

After the end of the follow-up, eGFR and UPCR were re-assessed, and we calculated the percentage change of eGFR and UPCR for each patient. In univariate regression models, all baseline recorded variables (see Table 1)—ox-LDL, UPCR, serum albumin, duration of T2DM and history of CV disease—were associated with the change of eGFR. Ox-LDL cholesterol was a strong modifier of the association between UPCR and eGFR decline in both univariate regression models (β = −0.001, *p* < 0.0001) and multivariate models (β = −0.05, *p* = 0.04), adjusted for ox-LDL, UPCR, albumin, T2DM duration and history of CV disease (Table 3), Figure 2.

As shown in Figure 2, the association between every 1 g/g increase in UPCR and eGFR decline over time was magnified as ox-LDL increased. That is, the unfavorable effect for eGFR decline portended by a fixed increase in UPCR (1 g/g) was progressively higher across increasing values of ox-LDL cholesterol.

## 4. Discussion

In our study 91 patients with proteinuric DKD, diabetic retinopathy and history of T2DM for at least 7 years were recruited. The main mechanism driving progression of proteinuric DKD is considered to be microangiopathy (in contrast to macroangiopathy for the non-proteinuric form). Having this in mind, our inclusion criteria included history of T2DM for at least 7 years, presence of proteinuria, eGFR below 90 mL/min and the presence of diabetic retinopathy. Our inclusion criteria to establish DKD, and not T2DM and CKD due to other causes, have been used in other previous studies [14,18,19]. At baseline, we divided our study cohort by eGFR tertiles, as described before [20,21], and found a progressive increase in inflammation and OS markers (CRP and ox-LDL) as eGFR decreased. As expected, the prevalence of proteinuria, malnutrition, anemia and uncontrolled hypertension gradually increased as eGFR decreased. After enrollment, we prospectively followed all participants with the primary outcome being a composite kidney outcome of progression to ESKD requiring renal replacement therapy or ≥30% reduction in baseline eGFR. After the end of the study, we re-evaluated eGFR and calculated the eGFR decline over time for each patient (secondary endpoint). These kidney outcomes have been repeatedly used as validated outcomes in various prospective studies in similar populations before [22,23,24]. Since the outcome of interest was the deterioration of kidney function, we could not monitor the progression of DKD in patients who died during the follow-up period. Therefore, we considered death as a competitive event, and we used in our analysis Fine and Gray sub-hazard models, which take into account the competitive event of death. Similar analyses with Fine and Gray models have been conducted in similar study settings and designs to ours [15,16,17,25].

During the follow-up period, 18.7% patients presented the primary kidney endpoint. Proteinuria was independently associated with both the primary outcome and with eGFR decline over time at the end of the study. Data from large epidemiological studies suggest that proteinuria is an independent predictor of DKD progression. The Chronic Renal Insufficiency Cohort (CRIC) Study enrolled 1908 participants with T2DM and decreased eGFR, and it found that 28% of them had some degree of proteinuria [24]. Proteinuric patients carried a much higher risk for DKD progression, development of ESKD and rapid decline in eGFR, compared to those without proteinuria. In another study, Vistisen et al. recruited 1984 T2DM subjects, followed them for up to 16 years, and found that the mean annual eGFR decline progressively increased across categories of albuminuria (1.9 mL/min in normoalbuminuria, 2.1 mL/min in microalbuminuria and 3.0 mL/min in macroalbuminuria) [26]. Although proteinuria plays a pivotal role in the pathogenesis and progression of DKD, through induction of tubular toxicity and interstitial scarring, its use as a biomarker for detection and prediction of DKD progression is characterized by high variability and lack of accuracy. Therefore, it is interesting to explore whether circulating biomarkers reflecting certain pathophysiologic changes might predict DKD progression. During the past decade, OS [27,28,29] and ED [30,31] have emerged as novel molecular pathways underlying the development and progression of DKD. Hyperglycemia and uremia trigger overproduction and accumulation of advanced glycation end-products (AGEs) and reactive oxygen species (ROS). These highly reactive molecules upregulate pro-inflammatory and fibrogenetic pathways, cause significant oxidative modifications in the structure and function of proteins and lipids and trigger direct endothelial injury, through nitric oxide depletion, leading to oxidation of LDL cholesterol within the arterial wall [6]. Oxidation of LDL cholesterol, the hallmark of ED and atherosclerosis, has been repeatedly associated with adverse CV outcomes in various settings. Moreover, there is a growing body of evidence supporting that ox-LDL plays a pivotal role in the pathogenesis of DKD. In experimental hyperglycemic conditions, ox-LDL is overproduced and is excessively up-taken by several types of renal cells including epithelial, tubular, mesangial cells and podocytes, leading to kidney fibrosis, glomerulosclerosis and eventually loss of kidney function [32,33,34,35]. The finding that kidney accumulation of ox-LDL is associated with interstitial fibrosis and glomerulosclerosis was also verified in a biopsy study in kidney transplant recipients [36]. Ox-LDL, along with inflammatory and fibrotic factors (such as vascular endothelial growth factor, interleukin-6 and tumor necrosis factor a), exert deleterious effects on vascular permeability and kidney blood flow, resulting in the development of proteinuria [37]. Moreover, both systemic and kidney hyperglycemia cause an increase in asymmetric dimethylarginine (an endogenous inhibitor of nitric oxide synthase), resulting in endothelial injury, inflammation, fibrosis, OS, formation of ox-LDL and, eventually, in the development of albuminuria [30,31], whereas experimental data suggest that reduction in kidney asymmetric dimethylarginine, through improvement of OS and ED, is accompanied by a significant amelioration of diabetic nephropathy [30,31]. In proteinuric DKD, the traditional progression pattern included the development of proteinuria as a first, obligatory step and then a gradual deterioration of kidney function. Since ox-LDL represents a pathophysiologic pathway that results in the development of proteinuria, it became evident that these two biomarkers might interact in determining the increased risk for deterioration of kidney function in DKD. As expected, in our study, we found that ox-LDL modifies the UPCR–DKD progression link by amplifying the risk for deterioration of kidney function portended by progressively increasing levels of UPCR. However, this interaction was lost after adjustment for various co-founders, such as eGFR, SBP, serum albumin and duration of T2DM. On the other hand, ox-LDL also modified the association between UPCR and eGFR decline rate, even after adjustment for several co-founders, including duration of T2DM, serum albumin and background history of CV disease.

To our knowledge this is the first research study reporting an effect modification by ox-LDL on the association between UPCR and DKD progression in proteinuric DKD. Traditionally, epidemiologic studies aim to investigate whether a certain risk factor is causally related with the occurrence of a certain disease. However, it has been documented that causal risk factors might not have the same impact under all conditions. Their effect might be modified (either magnified or minimized) by other factors, implicated in their pathophysiologic pathway. This phenomenon is termed as interaction or effect modification in research medicine [16] and has been examined in various clinical studies. It has been reported that adiponectin modifies the association between resistin and mortality in ESKD patients [38], whereas in another prospective study from our group, we reported a mutual effect modification between adiponectin and HDL cholesterol as risk factors for CV events in T2DM patients [15]. The effect modification phenomenon has also been studied for ox-LDL. In a recent study with quite similar statistical analysis, Spoto et al. investigated whether ox-LDL modifies the association between serum gamma-glutamyltransferase and mortality in a cohort of 1038 elderly individuals, and they found that ox-LDL amplified the magnitude of this link [39].

The finding in our study, that ox-LDL and UPCR are intra-related entities, is in keeping with the hypothesis of a role of these two biomarkers in the development and progression of DKD. Cross-sectional data suggest that among T2DM patients divided into normo-, micro- and macro-albuminuric, there is a graded increase in ox-LDL circulating levels, regardless of glycemic control [10,11]. Moreover, the prospective Diabetes Control and Complications Trial showed that ox-LDL particles exert pro-inflammatory properties and play a key role in the development of proteinuria in type 1 diabetes subjects [12].

Our study has certain limitations that should be addressed, including the low sample size and the observational design, which precludes establishing any causality. As in any observational study, although we adjusted our models for several factors, our results might have been influenced by other unidentified confounders. Moreover, we did not re-assess ox-LDL levels at the end of the study and the number of patients in each group was too small to draw definite conclusions on the examined associations. Despite these limitations, this study supports that there might be an interplay between ox-LDL and UPCR in the pathogenesis of DKD. Future larger, well-designed studies with larger sample size assessing a broader range of parameters in other DKD patterns (both proteinuric and non-proteinuric) are needed in order to draw definite conclusions regarding the possible interaction between ox-LDL and proteinuria as predictors of DKD progression.

In conclusion, our study shows that UPCR is independently associated with progression of DKD and deterioration of kidney function in proteinuric DKD, and that circulating ox-LDL levels might modify this association. The pathophysiology of the link between ox-LDL-UPCR and progression of DKD requires further investigation, but this observation suggests a role of UPCR in the deleterious effect of ES and ED on progression of DKD.

## Figures and Tables

**Table 1:** Baseline anthropometric, clinical and biochemical data of patients with proteinuric DKD according to tertiles of estimated glomerular filtration rate. Results for continuous variables are presented as mean (S.D.) or median (range).

	Estimated Glomerular Filtration Rate (mL/min/1.73 m^2^)				
	**All Subjects** **(*n* = 91)**	**Tertile 1** **(*n* = 30)**	**Tertile 2** **(*n* = 31)**	**Tertile 3** **(*n* = 30)**	***p***
eGFR (mL/min/1.73 m^2^)	59.6 (18–89.6)	33.1 (18–41.3)	59.6 (42–72)	83.3 (73.3–89.6)	**<0.0001**
Age (years)	67 (47–84)	71 (47–81)	70 (50–84)	63 (50–78)	**0.026**
Gender, Male (%)	47	63	42	36	0.09
History of CV events (yes, %)	70.3	83.3	71	56.7	0.08
Duration of T2DM (years)	13 (7–35)	16 (7–35)	13 (7–28)	10.5 (7–26)	**0.05**
Duration of hypertension (years)	13.0 (2–42)	16.5 (3–34)	17 (3–42)	12 (2–25)	0.09
Waist circumference (cm)	106.4 (12.3)	106.1 (11.6)	108.8 (13.4)	104.3 (11.9)	0.36
SBP (mm Hg)	140 (100–180)	145 (100–180)	140 (120–180)	130 (120–165)	**0.024**
DBP (mm Hg)	80 (50–95)	80 (50–95)	80 (60–95)	75 (60–90)	0.12
Hemoglobin (g/dL)	12.6 (1.7)	11.8 (1.8)	13.0 (1.2)	12.9 (1.6)	**0.011**
Fasting Glucose (mg/dL)	157.8 (50.9)	165.4 (68.2)	150.5 (36.9)	157.7 (43.2)	0.86
HbA1c (%)	7.2 (5.0–11.6)	7.6 (5.7–11.6)	7.1 (5.0–10.8)	7.2 (6.3–10.7)	0.69
Albumin (g/dL)	4.3 (3.2–5.0)	3.9 (3.2–5.0)	4.4 (3.8–4.8)	4.3 (4.0–4.9)	**0.009**
Total cholesterol (mg/dL)	176 (103–345)	171 (112–240)	177 (128–300)	174 (103–345)	0.62
LDL cholesterol (mg/dL)	94.5 (41–245)	96.5 (52–157)	95.5 (64–206)	92 (41–245)	0.81
HDL cholesterol (mg/dL)	47 (27–105)	42 (29–59)	48 (27–84)	49 (31–105)	**0.04**
Triglycerides (mg/dL)	140 (52–450)	180.5 (59–450)	164 (66–292)	100 (52–320)	**<0.0001**
Oxidized LDL (U/L)	66.2 (22.9–123.4)	72.2 (33.2–96.7)	70.1 (45.4–105.3)	53.8 (22.9–123.4)	**0.022**
UPCR (g/g)	0.15 (0.007–6)	0.5 (0.007–6)	0.14 (0.02–1.6)	0.1 (0.01–0.98)	**<0.001**
CRP (mg/dL)	0.2 (0–11)	0.31 (0–2.6)	0.2 (0–11)	0.1 (0–4)	**0.004**

*p* values of independent t-test, Mann–Whitney U or ANOVA test for differences of variables and χ2 test for differences in frequencies among eGFR quartiles. In bold, *p* values < 0.05. eGFR, estimated glomerular filtration rate; CV, cardiovascular; T2DM, type 2 diabetes mellitus; SBP, systolic blood pressure; DBP, diastolic blood pressure; HbA1c, glycated hemoglobin A1c; LDL, low-density lipoprotein; HDL, high-density lipoprotein; UPCR, urine protein-to -creatinine ratio; CRP, C-reactive protein.

**Table 2:** Cox proportional analysis (Fine–Gray sub-distribution hazard model) showing predictors for the composite kidney outcome of eGFR decline over 30% from baseline or progression to end-stage kidney disease requiring dialysis in patients with proteinuric DKD.

eGFR Decline over 30% from Baseline or Progression to ESKD		
	Crude model	Adjusted model
Variables (units of measurement)	SHR (95% CI), p	SHR (95% CI), p
**Ox-LDL × UPCR interaction(U.L/g.g)** Adjusted for the main effect of UPCR and Ox-LDL	1.01 (1.00–1.01), ***p* < 0.001** (see Figure 1)	1.01 (0.98–1.04), *p* = 0.4
**Ox-LDL (U/L)**	1.05 (1.02–1.08) ***p* < 0.0001**	1.07 (1.03–1.12) ***p* = 0.002**
**UPCR (g/g)**	1.53 (1.15–2.03) ***p* = 0.003**	0.58 (0.06–5.9) *p* = 0.65
**eGFR** **(mL/min/1.73 m^2^)**	0.97 (0.95–0.99) ***p* = 0.03**	0.99 (0.96–1.04) *p* = 0.96
**SBP (mm Hg)**	0.96 (0.93–0.99) ***p* = 0.01**	0.97 (0.94–0.99) ***p* = 0.05**
**Duration of T2DM (years)**	1.05 (1.00–1.11) ***p* = 0.03**	1.07 (0.99–1.17) *p* = 0.09
**Serum albumin (g/dL)**	0.25 (0.11–0.60) ***p* = 0.002**	0.99 (0.95–1.05) *p* = 0.97

eGFR, estimated glomerular filtration rate; ESKD, end-stage kidney disease; SHR, sub-hazard ratio; CI, confidence interval; Ox-LDL, oxidized low-density lipoprotein cholesterol; UPCR, urine protein-to-creatinine ratio; SBP, systolic blood pressure; T2DM, type 2 diabetes mellitus.

**Table 3:** Regression coefficients (β) of multiple regression models, with the dependent variable as the percentage change of eGFR over time and independent variables selected on the basis of respective associations.

	*β*	Standard Error	*p*
**Model 1**			
Ox-LDL × UPCR interaction	−0.001	0.003	**<0.0001** **(see Figure 2)**
**Model 2**			
Ox-LDL × UPCR interaction	−0.05	0.002	**0.04** **(see Figure 2)**
Ox-LDL	−0.003	0.001	0.08
UPCR	0.30	0.17	0.08
Duration of T2DM	−0.004	0.003	0.24
Serum albumin	0.02	0.07	0.81
History of CV disease	0.004	0.06	0.94

Model 1: Unadjusted. Model 2: Adjusted for all variables associated with eGFR change over time in unadjusted models (Ox-LDL, UPCR, duration of T2DM, serum albumin and background history of CV disease). Ox-LDL, oxidized low-density lipoprotein cholesterol; UPCR, urine protein-to-creatinine ratio; T2DM, type 2 diabetes mellitus; CV, cardiovascular.

## Data Availability

All data used to support the findings of this study are included within the article.
